# The role of advocacy and empowerment in shaping service development for families raising children with developmental disabilities

**DOI:** 10.1111/hex.13539

**Published:** 2022-05-29

**Authors:** Zsofia Szlamka, Bethlehem Tekola, Rosa Hoekstra, Charlotte Hanlon

**Affiliations:** ^1^ Department of Psychology Institute of Psychiatry, Psychology and Neuroscience, King's College London London UK; ^2^ Department of Health Services and Population Research Centre for Global Mental Health, Institute of Psychiatry, Psychology and Neuroscience, King's College London London UK; ^3^ Department of Psychiatry, School of Medicine, WHO Collaborating Centre for Mental Health Research and Capacity‐Building, College of Health Sciences Addis Ababa University Addis Ababa Ethiopia; ^4^ Global Health, Centre for Innovative Drug Development and Therapeutic Trials for Africa (CDT‐Africa), College of Health Sciences Addis Ababa University Addis Ababa Ethiopia

**Keywords:** advocacy, developmental disabilities, developmental disorders, empowerment, rights‐based approaches, service development

## Abstract

**Introduction:**

Empowerment of families raising children with developmental disabilities (DDs) is essential to achieving rights‐based service development.

**Methods:**

In this qualitative study, we investigated stakeholder perceptions on the role of advocacy and empowerment in developing caregiver interventions for families of children with DDs in a global context. Participants had experience with at least one intervention, namely the Caregiver Skills Training developed by the World Health Organization (WHO). Participants were clinicians, caregivers and researchers representing five continents, and representatives of WHO and Autism Speaks. Two focus group discussions and 25 individual interviews were conducted. Data were analysed thematically.

**Results:**

Three themes were developed: empowerment as independence and as a right; the role and practices of advocacy; and using evidence to drive advocacy. Many professional participants defined empowerment within the realms of their expertise, focusing on caregivers' individual skills and self‐confidence. Caregivers expressed that this expert‐oriented view fails to acknowledge their intuitive knowledge and the need for community‐level empowerment. Participants discussed the challenges of advocacy in light of competing health priorities. The gap between the rights of caregivers and the availability of services, for example, evidence‐based interventions, was highlighted as problematic. Scientific evidence was identified as a key for advocacy.

**Conclusion:**

Rights‐orientated empowerment of caregivers and advocacy may make vital contributions to service development for children with DDs in contexts worldwide.

**Patient and Public Contribution:**

Research questions were revised based on views presented during focus group discussions. Participant feedback on preliminary themes informed the development of the interview guides.

## 1 INTRODUCTION

Caregivers of children with developmental disabilities (DDs), including autism and intellectual disability, often struggle to claim the rights of their child, such as access to health and social and financial support.^1^ Empowerment approaches aim to address this struggle, despite critiques that the term empowerment lacks clear definitions and meaning.^2^ Empowerment can take place on an individual, organizational and community‐based level.^3^ Empowerment approaches often rely on experts empowering beneficiaries. These approaches may mean psychological empowerment that focuses on the skills and resources of an individual,^4^ community‐based empowerment highlighting societal power imbalances^5^ and economic empowerment targeting the economic well‐being of individuals and communities.^6^ An example is the World Health Organization's (WHO) community‐based rehabilitation guidelines that identify empowerment as one of the five key components of improving the lives of persons living with disabilities.^7^ WHO's definition of empowerment encompasses advocacy and communication, community mobilization, engaging with self‐help groups, political participation and representation in Disabled People's Organizations.^7^

Evidence from well‐resourced settings indicates that caregivers rely on their educational and professional backgrounds to advocate and access services.^8^ Meanwhile, in lower‐income settings advocacy groups of self‐advocates or caregivers might not exist or there might only be a few of them.^9^ When they exist, they often focus on local communities instead of a national or continental advocacy campaign.^10^ Numerous barriers, such as low literacy and lack of internet, may limit access to advocacy‐related information.^10^ Literature from both low and higher resource settings suggests that barriers to being an advocate include cultural differences between health providers and caregivers,^1^ or caregivers feeling uncomfortable speaking up publicly.^11^ The socioeconomic status of caregivers can pose further limitations in advocating for their children because of work schedules, financial resources and sometimes a lack of access to information about their children's rights.^12^

Caregiver advocacy is described as a set of behaviours, including obtaining support or services for the child, being a voice for a child, creating opportunities, facilitating change and educating the community about the child's needs.^1^ Similar to empowerment, the concept of caregiver advocacy has been inconsistently defined in the literature. Some scholars focus on what the key goal of advocacy is, suggesting for example to achieve social justice.^13^ Meanwhile others define advocacy as such that it has overlapping elements with the definitions of participation and empowerment.^14^ Rights‐based approaches give a theoretical grounding to advocacy for DDs.^15^ These approaches originate from the aim of implementing a human rights framework in health: so that access to services and empowerment, as human rights, should serve as key principles for the provision of care.^15^ From the perspective of disability inclusion, it means that persons with disabilities should be able to enjoy citizenship on an equal basis with others.^16^

Caregivers often describe advocacy as one of the many roles they play, alongside being carers, partners, and educators.^17^ Active engagement in advocacy, including for service development, is valued by caregivers as a constructive way to respond to the difficulties they face.^18^ Based on the idea that caregivers are essential in developing services for DDs, there are examples of training interventions for caregivers to become advocates for services,^19^ for example, for Latino families in the United States.^20^ Family peer advocates have been shown to be effective in facilitating service access for minority groups, for example among black and Hispanic caregivers of children with autism.^21^ Engaging in advocacy is shaped by caregiver access to cultural^14^ and social capital^14^ and resources.^14^ Existing literature outlines conceptual challenges with empowerment and advocacy, and core empowerment frameworks rely on expert views. Little is known about the diversity of understandings regarding empowerment and advocacy for families with DDs.

### 1.1 Aims

The aim of this study was to investigate advocacy and empowerment in relation to service development for families of children with DDs in low‐ and middle‐income country settings. We studied the following research questions:

1. How is empowerment understood among stakeholders supporting families with a DD, or raising children with a DD?
2. How do stakeholders perceive the role that advocacy and empowerment play in service development for DD?
3. What are the ways in which evidence is used for advocacy and empowerment for DDs?

## 2 METHODS

This qualitative study took a phenomenological approach to focus on the individual experiences of stakeholders (25). The study questions were applied to general service development for caregivers of children with a DD and to a specific caregiver intervention, the WHO's Caregiver Skills Training (CST). This programme was chosen as it represents a global effort to make services available for caregivers irrespective of their socioeconomic setting. Besides, there is a wide range of stakeholders involved in its adaptation and implementation, including caregivers and advocacy organizations.^22^ The CST grew out of a collaboration between WHO with Autism Speaks (a leading nongovernmental organization for autism research and advocacy) and international partners.^23^ CST is a low‐intensity training intervention for caregivers of children between 2 and 9 years with DDs or in whom a developmental delay has been identified but who have not yet received a formal diagnosis. The programme is evidence‐informed and consists of nine group sessions and three home visits.^22^

First, two focus group discussions (FGDs) were conducted during a technical consultation meeting of the WHO CST in Xiamen, China on 8–9th November 2018. This event was attended by country teams adapting and implementing CST and leads of CST in WHO and Autism Speaks. Overall, 15 participants attended the FGDs, 10 in the first and 5 in the second group. The FGDs focused primarily on adaptations of caregiver interventions and the findings have been presented in a separate paper.^24^ Empowerment and advocacy were identified as preliminary themes in this first part. Following on from the FGDs, 25 semi‐structured individual interviews were conducted. Interviews took place online, using a video chat software convenient to participants. Twenty‐three interviews took place in English and two in Spanish. The interviews focused on further exploring the preliminary themes developed using data from the FGDs.

### 2.1 Patient and public contribution

This study was initiated following reflections on conversations with caregivers who participated in the local adaptation of CST and with CST team members in different countries. The views shared by stakeholders during the initial FGDs also framed the final research questions and informed the topic guides for the in‐depth interviews that followed. Preliminary themes developed based on the analysis of FGDs were summarized and presented to FGD participants in the form of a summary document. Participants had the opportunity to provide their feedback on these preliminary findings. Their feedback informed the development of the interview guides for individual interviews.

### 2.2 Participants

Study participants were included in the study if they were clinicians, researchers, caregivers of children with a DD or representatives of WHO or Autism Speaks. A further inclusion criterion was that participants had previous experience with caregiver interventions. For the FGDs, potential participants had to be present during the WHO CST consultation meeting in Xiamen. Potential participants were excluded from the study if they did not speak English or Spanish. Interviewing WHO and Autism Speaks representatives helped data source triangulation as it provided a global perspective on the research questions.^25^ For details of FGD participants see Table 1; for interview details see Table 2. Participants did not receive financial incentives to participate in the study.

**Table 1:** Characteristics of participants in focus group discussions

Region	Overall	Clinicians	Caregivers	Researchers
*FGD1*				
Africa	**1**			1
Americas				
North America	**0**			
Central and South America	**4**	2	2	
South‐East Asia	**1**	1		
Europe	**0**			
Eastern Mediterranean Region	**0**			
Western Pacific	**4**	2	1	1
*FGD2*				
Africa	**0**			
Americas				
North America	**1**			1
Central and South America	**4**	3	1	
South‐East Asia	**0**			
Europe	**0**			
Eastern Mediterranean Region	**0**			
Western Pacific	**0**			

**Table 2:** Characteristics of participants in individual interviews

Region	Overall	Clinicians	Caregivers	Researchers	WHO/AS
Africa	2	1		1	
Americas					
North America	4				4
Central and South America	11	6	3	1	1
South‐East Asia	1	1			
Europe	1	1			
Eastern Mediterranean Region	1				1
Western Pacific	5	3		2	

### 2.3 Interview guides

The interview guide for FGDs and individual interviews were both designed based on previous literature on empowerment.^4^, ^26^, ^27^ The interview guide was adjusted and further refined iteratively after each interview. A separate topic guide was developed for participants from WHO and Autism Speaks. Along with interviews, contextual information and reflections on the main topics were noted in an interview diary. All interview guides can be found in the Supporting information.

### 2.4 Analysis

Following transcription by Zsofia Szlamka, data were anonymized and analysed using thematic analysis in NVivo 12.^28^ Thematic analysis was chosen because of its flexibility and the exploratory nature of the study.^29^ Themes were developed iteratively following discussions between all authors, allowing for investigator triangulation.^25^ First, inductive coding strategies were used to develop preliminary themes based on the FGDs. This preliminary codebook served as a starting point for analysing the individual interviews. Analysis was also informed by literature on the exercise of power in global health, equity in global health partnerships^30^ and empowerment of families with DDs.^4^, ^31^ Therefore, deductive coding was used to identify the role of power and stakeholder partnerships in the data. For example, theme one on empowerment was a result of deductive coding. Once main themes were developed, they were compared across regions and stakeholder groups.

## 3 RESULTS

The following key themes were developed from the data and informed by literature: (a) Empowerment as independence and as a right, (b) the role and practices of advocacy and (c) using evidence to drive advocacy. Each theme will be discussed in detail, with illustrative quotes. All additional relevant quotes can be found in File S1.

### 3.1 Empowerment as independence and as a right

Informants reported that empowerment should concern all family members and allow for the person with a DD to develop their full potential. All participants thought that the empowerment of families with DDs is a key aspect of service development. However, professionals and caregivers had different perspectives on what empowerment means and what outcomes can be expected from it.

Most professional participants shared the view that empowerment starts with changing attitudes. Some gave the example that if caregivers learn more about DDs as part of an intervention, they could understand what their children could achieve. They could also involve them in suitable tasks and responsibilities.

3.1.1

*We were able to empower these parents…because those parents did not think that children with disabilities are supposed to be given tasks*. PCP18, interventionist, African Region

Many informants working with families with DDs defined empowerment outcomes within their area of expertise. They had a strong focus on the child with DD and the family and less emphasis on wider societal action. For example, participants working in governments or disability organizations thought that a key aspect of empowerment is being able to grow financially and have income‐generating activities. Meanwhile, some clinicians explained that an empowered caregiver can better improve the child's social and communicational skills. Other clinicians reported that empowerment means developing a stronger relationship between caregivers and children.

3.1.2

*When I say empowerment, I mean caregivers having more skills, more tools, more knowledge and thus having more power…to influence and to impact positively their son's or daughter's development. So, when we talk about empowerment, we mean caregivers learning how to better promote development in their kids*. PCP4, clinician, Americas Region

A view that professional participants shared was that being empowered has an element of self‐care and self‐confidence. Informants thought that an empowered caregiver is confident, feels useful in society, does not feel ashamed and takes care of oneself. They added that the empowered caregiver has the relevant knowledge to support the child with a DD and decreases the need to receive help from others. Some participants pointed out that empowerment may be defined differently across contexts. They suggested that goals such as relying on oneself might be alien to certain communities. Others suggested that families in different countries may be empowered to a different extent due to contextual differences between countries. An example they added was that caregivers in higher‐income settings like the United States of America may have more opportunities for empowerment and advocacy. They also thought that factors such as acceptance of a disability in a family, civil society mobilization and the availability of advocacy organizations for DDs in a setting can facilitate empowerment.

3.1.3

*I saw in United States, there is very common for families to be very empowered and have an institution and have their own government, like a lobby, to get things and […] to get laws […] and here in our country this is very new and we need to educate the people to get these things [advocacy organizations lobbying for new policies]*. PCP16, clinician, Americas Region

Professional participants had different views regarding what format empowerment may take. Some saw empowerment as a set of steps and milestones that caregivers might achieve. Many clinicians thought that being in a group of caregivers can be empowering. They believed that group dynamics can reduce their stress levels while experiencing a higher level of power, leading to broader advocacy for change. Others saw it as a journey:

3.1.4

*We've been looking at the journey of parents and they start to implement some of the strategies and amplify what they have done, generalising it to different areas of their work and then finally coming to a stage where they are advocating for their children, not just for themselves but for other children, creating better services, pushing for better services, legislature, policies, changes in the communities, that's a long journey, that's what we see from the parents*. PCP5, clinician, South‐East Asia Region

Caregivers thought that empowerment is about rights and control over their decisions. They discussed that their expertise by experience and intuitive knowledge about supporting their children can be used to work towards the inclusion of children with DD in society. They shared the view that caregivers can play a crucial role in advocating for service development and closing the gap between rights and services available. They shared that a key pillar of their work is parent‐to‐parent mentoring and peer support. In this process, caregivers suggested they learn how to take an active role in helping their children.

3.1.5

*…what we intuitively know…parents, we know. So we don't know how, but we know. So it's very important that they [professionals] teach us how to, but what we do intuitively…I have personally been doing that for 10 years without a format when we started a parents group, and parents learn from older parents how to and then they do the same with the younger*. PCP2, caregiver and NGO representative, Americas Region

Many participants from all stakeholder groups involved observed a discrepancy between the rights of caregivers and what is practically available in many contexts. Caregivers thought that this incongruence has a large impact on their life in many areas and they mentioned access to education as an example. Despite the fact that the inclusion of persons with disabilities in education is a right outlined by the law in many countries, there are no schools available for children with DDs. Moreover, there is a lack of trained teachers who would know how best to include children with DDs.

3.1.6

*When school says I cannot have your child and they are breaking the law in the face of the mother and the mother does not know the law and they are trespassing all the human rights of the children…So you have to empower parents, not only from helping how to be in bathroom, but also know that your right is…For example a school says okay, I cannot accept you but I will make you a favour and accept you. You have to know that that is a lie. And maybe you can say okay, and shut up because you decide to shut up, because that's the only school available at the moment and something very different is to believe that it is right what they are telling you*. PCP2, caregiver and advocate, Americas Region

Caregiver participants added that the reason why caregiver empowerment is especially needed is to address this gap between their theoretical rights and services available in practice. Beyond this, many caregivers reported that empowerment can also help more caregivers become advocates for service development, develop the service themselves and take an active role in supporting their children.

3.1.7

*We won't wait [for] the government, we are gonna [going to] do it by ourselves, so you have to make a step, and go to the training and you will help yourself through us*. PCP14, caregiver and NGO representative, Americas Region

### 3.2 The role and practices of advocacy

Participants from all stakeholder groups described the key features and challenges of advocacy in light of competing health priorities. Informants involved in local advocacy thought that civil society is key to advocating for service development for DDs by the government. Others added that civil society can contribute to the design of free and accessible services tailored for caregivers of children with DD. Some thought that advocacy and awareness‐raising should also be the responsibility of service providers.

3.2.1

*If we leave everything in the government's hands, nothing will happen, as part of the civil society we insist that it [caregiver intervention] is applied and utilised*. PCP10, caregiver and advocate, Americas Region

Informants thought that one of the key areas where advocacy can support service development is pushing for having more services available. Most participants agreed that practitioners cannot always choose which intervention to use based on available scientific evidence. Rather, they often choose any intervention that is available in their setting.

3.2.2

*It's always a bit related to chance and possibilities there are in a country, to determine the programme. Or if there is a training of that programme in your country and there are people who have been trained in it…generally it's not based in okay which ones are evidence‐based and let's go with the evidence‐based things, no, it's what do we have at hand and let's go with that and we will try out that, mostly*. PCP4, clinician, Americas Region

Advocacy can help so that DDs are considered health priorities, show how DD affects areas like education and gain visibility among policymakers. Overall, participants maintained that personal connections, media communication and taking advantage of opportunities play a role in raising awareness of DDs in a community.

3.2.3

*Look, in our country, things work through other people. I talked about [DDs] on a TV channel, in a journal, on Twitter, in a Facebook story, so that other people, stakeholders, like the politicians react to it. We have to be very persistent to get to them*. PCP10, caregiver and advocate, Americas Region

Informants described practices that can help advocate for DDs. Some thought that gaining support from people in leadership positions, for example, in government, can help advocacy. Others added that cooperating with well‐known international organizations can help buy‐in from governments and civil society for an intervention. Some mentioned that in certain settings the cooperation of a well‐known organization is necessary to receive governmental support. Finally, they added that the need to develop services for DDs can be approached from different perspectives, for example, from that of gender. In contexts where caregiving is primarily done by women, initiating a caregiver intervention could take place through cooperation with women's organizations.

3.2.4

*In this country, it is good to be their [government] friends…because you basically can't have an NGO without some kind of government support…it's [advocacy] geared towards disability but it's really coming from a women's perspective and the main driver for the fact that the country wants a women's foundation to be involved in this work is because whenever you have a child diagnosed with a disability like autism in the family, it's usually the women who quit their job*. PCP23, international organization representative

Participants mentioned the challenges of advocacy. Examples they mentioned include conflicting organizational interests that cause delays in service development. Another example they added is segmented organizations, where colleagues in related fields do not work closely together. This means that it is harder for the advocate to make sure all relevant stakeholders take part in service development.

### 3.3 Using evidence to drive advocacy

While changes may happen by chance or due to opportunism, participants did also see a role for scientific evidence in supporting advocacy. They mentioned that when talking about evidence, there are at least two types of data that can help the work of advocates: country‐specific prevalence rates of DDs and data regarding intervention outcomes. Some informants pointed to the lack of prevalence data in certain countries and suggested that this makes it more difficult to push for the development of support services. Perceptions of what it means if an intervention is evidence‐based ranged widely across participants: from having run a randomized control trial to observing changes on a child‐by‐child basis. Participants discussed that data can be used in support of different goals. Some thought that such goals can be receiving funding, gaining buy‐in from local government or convincing caregivers to choose and attend an intervention. A few informants noted that an evidence base for caregiver interventions is a tool for advocacy, helping the initiation and funding of an intervention. They thought that data regarding the local implementation of the intervention was the most effective data to best support advocacy.

3.3.1

*In our countries, in LMICs, you know there is almost nothing done in research. So when you have the evidence and you can show to government agencies and say you know we did this, and look what happened, so we need more funding, so it's like a presentation card. Like the invitation letter…it is not done in the UK, it is not done in the US, it is done in that country, so it works, you know?*. PCP24, international organization representative

Other informants also added that having an evidence base for a programme may not be relevant for all stakeholders. Policymakers may require data to support a programme. However, caregivers, especially in lower resource settings, might accept the help they can access and that meets their sociocultural context, without asking for evidence.

3.3.2

*When you talk to policy makers, being able to say that it [the intervention] is supported by science or informed by science, makes a huge difference…whereas families, especially in low‐resource settings, whether it's evidence base or not, may or may not make that big of a difference. I think for many families just having access to something, that's supposed to work, is a major step forward for them*. PCP23, international organization representative

## 4 DISCUSSION

In this study, we investigated the role that empowerment and advocacy play in shaping service development for families raising children with DDs. Overall, three key themes were developed: empowerment as independence and as a right, the role and practices of advocacy, and using evidence to drive advocacy. Our work adds to the existing literature by revealing differing understandings of empowerment for DDs.

The main theme that participants discussed was what empowerment means to them and what outcomes they expect from an empowering process. Professional participants defined empowerment within the realms of their expertise and tended to focus on empowerment at the individual level. They tended to focus on the situation of the caregiver and child with DD, for example, as having skills to improve the child's symptoms or as economic empowerment. Some suggested training caregivers on such skills, sometimes suggesting ‘correcting’ their attitudes towards DDs. The idea that empowerment takes place by offering information and education to caregivers is not without risks. Those delivering educational programmes, such as international agencies or highly trained professionals, are often able to do so because their knowledge is given more value due to their position and their organization's power.^32^ Therefore, they may exclude the voices and give insufficient weight to the intuitive knowledge of caregivers and expertise by lived experience. On the other hand, when caregivers cannot access information this risks entrenching marginalization. Those who lack services the most may lack the power to ask for what they need most, for example, evidence‐based interventions, inclusive law or education.

In the view of many professional participants, empowerment is also about independence and self‐confidence: being empowered means being able to take care of oneself. Many of them thought that an empowered caregiver relies less and less on others and is knowledgeable about how best to help his/her child with DD. This view of empowerment is strongly related to Western values of individualism, whereby dependence is understood as potentially harmful. These results are aligned with how psychological empowerment is defined in the existing literature: with a heavy focus on individual characteristics and skills.^4^ This expert‐oriented view offers a deficit‐oriented view of DDs and it does not acknowledge rights as a primary aspect of empowerment. This perspective misses the point as to what caregivers think they need, and how they define empowerment and it does not acknowledge their intuitive knowledge regarding DDs. Caregiver participants emphasized the importance of a rights‐based approach. They suggested that being empowered means knowing what their rights are as a starting point. When caregivers know their rights, they are more likely to be aware of all their options. This then leads to them being in control of their own decisions, even in cases if services are largely unavailable in a particular context. These caregiver perspectives suggest that including information on caregiver rights in parent‐mediated interventions might be a useful approach.

Rights‐based, participatory approaches to empowerment may address this by focusing on the process of empowerment without presupposing the outcomes.^33^ ^,^ ^15^ In this way, empowerment may become a tool for caregivers to overcome marginalization and underpin effective advocacy for ‘what matters most’ to them and their children with DDs. Such an approach could be synergistic with more skills‐focused approaches such as CST. Existing examples include studies from global mental health, for example, empowering service users to engage in stigma reduction activities^33^ or in setting priorities to strengthen mental health systems.^34^

The differentiation between individual and community empowerment has been described in previous work.^35^, ^36^ Meanwhile, some of our participants thought that empowerment is a process in which both caregivers and professionals take part. This understanding of empowerment can bridge the gap between experts' and caregivers' perspectives as it defines empowerment as a process in which different stakeholders interact. In previous literature this journey from the diagnosis to being empowered was linked to advocacy: caregivers find it helpful to have their voices heard.^1^, ^37^ This interactive and community‐oriented view resonates with community‐based approaches to empowerment, focusing on shared power, knowledge and information.^5^

Informants agreed that caregiver and professional advocacy can play a key role in service development, indicating a participatory approach involving both experts and lay people.^38^ They took a goal‐oriented approach to advocacy, focusing on practical next steps of service development and less so on more abstract goals such as advocating for rights. Stakeholder groups involved in advocacy usually include self‐advocates, caregivers, other family members^39^ and health workers.^40^ Informants mentioned the difficulty of bringing different sectors and organizations together to work towards service development, a challenge more often encountered in global mental health advocacy.^41^ Many thought that using personal connections, buy‐in for interventions from people in positions of power and taking advantage of opportunities that arise are key techniques of advocacy. This reflects that many advocates lack access to positions of power. Meanwhile, the literature suggests that opportunism may also serve as a means of effective advocacy if coupled with a framework of longer‐term goals.^42^ Possible ways forward to improve the impact of advocacy are to connect organizations at the grassroots with one another^10^ and on a global scale to other stakeholders working in the field of DDs.^43^ Some strides in this direction are being made, for example, the African Autism Advocacy Leadership meeting organized by WHO and Autism Speaks in January 2020, which brought together African researchers, grassroots advocates and representatives from international agencies. These types of initiatives can support caregivers and health workers in grassroots organizations to have a voice in setting health priorities. Informants shared the idea that caregivers can be advocates themselves and can take an active role in shaping service development for DDs. They agreed that empowerment practices and caregiver interventions can facilitate this. Some thought that there is a gap between the rights of caregivers and services and professionals available and added that one of the goals of empowerment is to overcome this gap.

### 4.1 Implications and future research

Our participants suggested that evidence can be a useful tool for advocacy: practicing advocates may use existing evidence to gain interest from stakeholders, receive funding or convince caregivers to participate in interventions. While policymakers often require evidence to support an intervention, caregivers (both from high and low resource settings) may not be in the position to ask or know whether an intervention is backed by evidence. This shows inequality in access to information and to evidence‐based interventions, a phenomenon that health practitioners may find relevant to take into account when working in primary care. As some participants suggested, caregivers may not say no to a programme that is not evidence‐based and would accept any help they can access. This puts service providers in a position of power and responsibility since some caregivers are more likely to accept the help that may not be evidence‐based, support from someone less qualified or they might not be interested in the evidence base at all.

Based on the results of this study, we also suggest that further research is needed to develop caregiver‐oriented empowerment frameworks. Moreover, the inequity in the availability and access to evidence‐based practices across income settings may be further studied.

## 5 LIMITATIONS

The study is limited in terms of its geographical representation. Most participants came from the Americas, while regions such as the African or the European regions were not well represented. Besides, interviews were conducted, transcribed and translated by the first author, who is neither a native English nor a native Spanish speaker. This may have lost some of the subtlety of the idiomatic use of language. The first author's positionality influenced the ways in which participants shared information. For example, participants' discussions about scientific evidence in advocacy were driven by the first author's question about the use of evidence during the FGDs. The first author, who conducted the interviews, had previously met some of the informants: this created a stronger rapport between the researcher and those participants. Certain participants may have thought that the first author works as part of the global CST team. This perceived role of the first author may have had an impact on how much participants were willing to share and to what extent they were willing to disclose their experiences. Participants were all connected to the WHO CST programme, despite having experience with a range of other interventions. Having participants not involved in WHO CST could have added another layer of experiences to explore. Similarly, participants who are self‐advocates of DDs were also missing from this study. Because the WHO CST intervention targets young children, none of our informants were self‐advocates, and that means an important perspective is missing from our study. Finally, a further limitation of this study is the low number of caregivers in the FGDs compared to professionals.

## 6 CONCLUSION

Empowerment of caregivers of children with DDs and advocacy for the rights and service access for families can play a key role in developing services in an inclusive manner. However, stakeholders working with DDs define empowerment and its outcomes in different ways. Professionals focus on the realms of their area of expertise and psychological empowerment. Meanwhile, caregivers focus more heavily on their rights, having control over their decisions and using advocacy for service development. From a rights‐based perspective, it is essential that caregiver voices are taken into account when developing and scaling up interventions for DDs across contexts and resource settings. Needs assessment and participatory approaches could help in doing so. Advocacy is a key in service development and caregivers can play an active role in it. Stronger collaboration across stakeholders could support this advocacy work. There should be a stronger research and policy focus on understanding power imbalances among stakeholder groups in DDs: those in positions of power are more likely to have their knowledge and views represented in services, while the voices of others might be underrepresented.

## CONFLICTS OF INTEREST

The authors declare no conflicts of interest.

### ETHICS STATEMENT

The study received ethical approval from the Psychiatry, Nursing & Midwifery subcommittee of King's College London's College Research Ethics Committee (reference: RESCM‐18/19‐8447). All participants provided written informed consent and confidentiality was ensured.

## Supporting information

Supporting information.

## Data Availability

Data are openly available in the article and attached as Supporting Information.

## References

[1] Boshoff K , Gibbs D , Phillips RL , Wiles L , Porter L . Parents' voices: ‘why and how we advocate’. A meta‐synthesis of parents' experiences of advocating for their child with autism spectrum disorder. Child Care Health Dev. 2016;42(6):784‐797. 27445227 10.1111/cch.12383

[2] Batliwala S . Taking the power out of empowerment—an experiential account. Dev Pract. 2007;17(4‐5):557‐565.

[3] Bakker L , Van Brakel W . Empowerment assessment tools in people with disabilities in developing countries. A systematic literature review. Lepr Rev. 2012;83:129‐153. 22997690

[4] Cattaneo LB , Chapman AR . The process of empowerment: a model for use in research and practice. Am Psychol. 2010;65(7):646‐659. 20873882 10.1037/a0018854

[5] Spreitzer GM . Taking stock: a review of more than twenty years of research on empowerment at work. Handb Organ Behav. 2008;1:54‐72.

[6] Jiménez‐Solomon OG , Méndez‐Bustos P , Swarbrick M , et al. Peer‐supported economic empowerment: a financial wellness intervention framework for people with psychiatric disabilities. Psychiatr Rehabil J. 2016;39(3):222‐233. 27618459 10.1037/prj0000210

[7] World Health Organization . Community‐based rehabilitation: CBR guidelines. 2010. Accessed May 23, 2022. https://apps.who.int/iris/handle/10665/44405 26290927

[8] Taylor S , Wright AC , Pothier H , Hill C , Rosenberg M . It's like I have an advantage in all this: experiences of advocacy by parents of children with disabilities from professional backgrounds. J Soc Soc Welf. 2019;46:159.

[9] Samadi SA , McConkey R . Autism in developing countries: lessons from Iran. Autism Res Treat. 2011;2011:145359. 22937242 10.1155/2011/145359 PMC3420542

[10] Abubakar A , Ssewanyana D , de Vries PJ , Newton CR . Autism spectrum disorders in sub‐Saharan Africa. Lancet Psychiatry. 2016;3(9):800‐802. 27568261 10.1016/S2215-0366(16)30138-9 PMC6858850

[11] Jegatheesan B , Fowler S , Miller PJ . From symptom recognition to services: how South Asian Muslim immigrant families navigate autism. Disabil Soc. 2010;25(7):797‐811.

[12] Lalvani P . Parents' participation in special education in the context of implicit educational ideologies and socioeconomic status. Educ Train Autism Dev Disabil . 2012:474‐486.

[13] Holcomb‐McCoy C , Bryan J . Advocacy and empowerment in parent consultation: implications for theory and practice. J Couns Dev. 2010;88(3):259‐268.

[14] Trainor AA . Diverse approaches to parent advocacy during special education home—school interactions: identification and use of cultural and social capital. Remedial Spec Educ. 2010;31(1):34‐47.

[15] Mann SP , Bradley VJ , Sahakian BJ . Human rights‐based approaches to mental health: a review of programs. Health Hum Rights. 2016;18(1):263. 27781015 PMC5070696

[16] Browne M , Millar M . A rights‐based conceptual framework for the social inclusion of children and young persons with an intellectual disability. Disabil Soc. 2016;31(8):1064‐1080.

[17] Lutz HR , Patterson BJ , Klein J . Coping with autism: a journey toward adaptation. J Pediatr Nurs. 2012;27(3):206‐213. 22525808 10.1016/j.pedn.2011.03.013

[18] Ewles G , Clifford T , Minnes P . Predictors of advocacy in parents of children with autism spectrum disorders. J Dev Disabil. 2014;20(1):73.

[19] Taylor JL , Hodapp RM , Burke MM , Waitz‐Kudla SN , Rabideau C . Training parents of youth with autism spectrum disorder to advocate for adult disability services: results from a pilot randomized controlled trial. J Autism Dev Disord. 2017;47(3):846‐857. 28070786 10.1007/s10803-016-2994-z PMC5354969

[20] Burke MM , Magaña S , Garcia M , Mello MP . Brief report: the feasibility and effectiveness of an advocacy program for Latino families of children with autism spectrum disorder. J Autism Dev Disord. 2016;46(7):2532‐2538. 26944592 10.1007/s10803-016-2765-x

[21] Jamison JM , Fourie E , Siper PM , et al. Examining the efficacy of a family peer advocate model for black and hispanic caregivers of children with autism spectrum disorder. J Autism Dev Disord. 2017;47(5):1314‐1322. 28168677 10.1007/s10803-017-3045-0

[22] Salomone E , Reichow B , Pacione L , Shire S , Shih A , Servili C . Training caregivers to transform children's lives. Early Child Matters. 2018;127:74‐77.

[23] Salomone E , Pacione L , Shire SY , Brown F , Reichow B , Servili C . Development of the WHO Caregiver Skills Training Programme for developmental disorders or delays. Front Psychiatry. 2019;10:769. 31780960 10.3389/fpsyt.2019.00769 PMC6859468

[24] Szlamka Z , Tekola B , Hoekstra R , Hanlon C . Perspectives on the role of context in adapting caregiver interventions for children with developmental disorders. 2021. Accessed May 23, 2022. https://osf.io/3krft/

[25] Thurmond VA . The point of triangulation. J Nurs Scholarsh. 2001;33(3):253‐258. 11552552 10.1111/j.1547-5069.2001.00253.x

[26] Nachshen JS . Advocacy, stress, and quality of life in parents of children with developmental disabilities. PhD Thesis; 1999. Accessed May 23, 2022. https://knowledgecommons.lakeheadu.ca/handle/2453/3098

[27] Masterson S , Owen S . Mental health service user's social and individual empowerment: Using theories of power to elucidate far‐reaching strategies. J Ment Health. 2006;15(1):19‐34.

[28] Braun V , Clarke V . Using thematic analysis in psychology. Qual Res Psychol. 2006;3(2):77‐101.

[29] Nowell LS , Norris JM , White DE , Moules NJ . Thematic analysis: striving to meet the trustworthiness criteria. Int J Qual Methods. 2017;16(1):1609406917733847.

[30] Shiffman J . Knowledge, moral claims and the exercise of power in global health. Int J Health Policy Manag. 2014;3(6):297‐299. 25396204 10.15171/ijhpm.2014.120 PMC4226618

[31] Balcazar F , Suarez‐Balcazar Y . Promoting empowerment among individuals with disabilities. In: Bond MA , Serrano‐García IE , Keys CB , Shinn ME , eds. APA Handbook of Community Psychology: Methods for Community Research and Action for Diverse Groups and Issues. 2017:571‐585.

[32] Freire P . Pedagogy of the Oppressed. Bloomsbury Publishing; 1968.

[33] Rai S , Gurung D , Kaiser BN , et al. A service user co‐facilitated intervention to reduce mental illness stigma among primary healthcare workers: utilizing perspectives of family members and caregivers. Fam Syst Health. 2018;36(2):198‐209. 29902036 10.1037/fsh0000338 PMC6005191

[34] Abayneh S , Lempp H , Hanlon C . Participatory action research to pilot a model of mental health service user involvement in an Ethiopian rural primary healthcare setting: study protocol. Res Involv Engagem. 2020;6(1):1‐14. 31934350 10.1186/s40900-019-0175-x PMC6951014

[35] Laverack G . An identification and interpretation of the organizational aspects of community empowerment. Community Dev J. 2001;36(2):134‐145.

[36] Hur MH . Empowerment in terms of theoretical perspectives: exploring a typology of the process and components across disciplines. J Community Psychol. 2006;34(5):523‐540.

[37] Luong J , Yoder MK , Canham D . Southeast Asian parents raising a child with autism: a qualitative investigation of coping styles. J Sch Nurs. 2009;25(3):222‐229. 19364878 10.1177/1059840509334365

[38] Tritter JQ , McCallum A . The snakes and ladders of user involvement: moving beyond Arnstein. Health Policy. 2006;76(2):156‐168. 16006004 10.1016/j.healthpol.2005.05.008

[39] McCoy MS , Liu EY , Lutz AS , Sisti D . Ethical advocacy across the autism spectrum: beyond partial representation. Am J Bioeth. 2020;20(4):13‐24. 10.1080/15265161.2020.1730482 32208091

[40] Sabo S , Ingram M , Reinschmidt KM , et al. Predictors and a framework for fostering community advocacy as a community health worker core function to eliminate health disparities. Am J Public Health. 2013;103(7):e67‐e73. 10.2105/AJPH.2012.301108 PMC3682609 23678904

[41] Vigo DV , Patel V , Becker A , et al. A partnership for transforming mental health globally. Lancet Psychiatry. 2019;6(4):350‐356. 30704963 10.1016/S2215-0366(18)30434-6

[42] Nathan S , Rotem A , Ritchie J . Closing the gap: building the capacity of non‐government organizations as advocates for health equity. Health Promot Int. 2002;17(1):69‐78. 11847140 10.1093/heapro/17.1.69

[43] Murillo L , Shih A , Rosanoff M , Daniels AM , Reagon K . The role of multi‐stakeholder collaboration and community consensus building in improving identification and early diagnosis of autism in low‐resource settings. Aust Psychol. 2016;51(4):280‐286.

