# Supplementary material for: The role of advocacy and empowerment in shaping service development for families raising children with developmental disabilities
Source: Health Expect. 2022 May 29;25(4):1882–91. doi: 10.1111/hex.13539 (PMC9327816; doi:10.1111/hex.13539)
Supplement: Supplementary file 1 — Supporting information. [file HEX-25--s001.docx]

# Supporting information

**The role of advocacy and empowerment in shaping service development for families raising children with developmental disorders**

Why empowering families?

This document contains all relevant quotes for the manuscript titled “The role of advocacy and empowerment in shaping service development for families raising children with developmental disorders” by Szlamka et al.

| **Empowerment** | *PCP23, international organisation representative* | *“I think what CST does is that it connects it creates an avenue where we can take the parents the expertise, the knowledge to their children and channel in a way that can be leveraged that can promote better development, right, so that is empowerment, at its core. What I care about is…how the parent, caregiver, see, fundamentally see the difference, the change in their relationship to their children and that to me is empowerment.”* |
| --- | --- | --- |
|  | *PCP20, clinician, WPRO* | *“So like to help the child develop, to help their child to learn some like a language, some skills and they have that kind of competence, I think that’s the empowerment.”* |
|  | *PCP21, international organisation representative* | *“If you are full of shame, you are not gonna be empowered, if you have no skills and you feel useless, you’re not gonna be empowered.”* |
|  | *PCP15, clinician, PAHO* | *“I think it [empowerment] can be achieved with the [ac]knowledgement, first of all, and we gave for the individual as the cues, the strategies, as the way that CST proposed…and I think it makes the person be able to solve their problems, by themselves. And it’s very different from our culture. Because the people that we treat, always wait [for] the politicians, the service, the outsource somebody to take care of them… And they are passives, passive citizens, they are not critical participants.”* |
|  | PCP7*, clinician, WPRO* | *“It is a construction of many things, like construction of self-efficacy, omm a sense of trust towards themselves, the feeling of being accepted, omm do they feel supported despite of the circumstances, I think it takes quite a lot of things to empower somebody. And to do so, omm it actually takes a lot of soft skills I believe. Or not by soft skills but by a true respect towards another human being, I would say to empower.”* |
|  | *PCP18, interventionist, AFRO* | *“Another thing was introducing them [the caregivers] to the national council for persons with disabilities, it was also empowering for them to know that with this council they are able to get financial and social support…they can also be given an allowance as groups of parents who are living with children with disabilities so they can start different projects which would help them in the future to take care of their children with disabilities*.” |
|  | *PCP2, caregiver and advocate, PAHO* | *“There is something that is called innocent discrimination, and for example when you tie the shoelaces of your son, because poor him, he cannot do that, you are discriminating your own son. And you don’t know it. So that is to recognise that the other person is different from you…and maybe you do many things for love…you know when I was at the UN… every panel had an autistic person. And one of them, I will never forget, he said you call it protection, but it is oppression. You know? You call it overprotection but it is oppression.”* |
|  | *PCP4, clinician, PAHO* | *“When the parents are learning together in a group and they are sharing the experiences between them, something happens there too which I can not explain but it is very, very empowering…I’m talking about mood first… when they get sometimes to the workshop, they are sad, or angry or desperate or without hope and the effect of empowerment impacts on mood so they are happier and they are more relaxed, they are less stressed…and I think this has to do with an augmented power, basically.”* |
|  | *PCP21, International organisation representative* | *“You know we’re not teaching them advocacy skills, we are not encouraging them to become advocates, like it’s not a direct goal of the programme but I think […] it could be an indirect outcome.”* |
|  | *PCP3, clinician, PAHO* | *“They get more involved in whatever needs to be done and they can talk to others, with authority… then they can ask for things what they need, ask when they don’t understand, they can go to school and say to the director or the teachers whatever they think is better for their kids, so for me empowerment is that they are the owner of their decision, they can…advocate for whatever they need and they don’t stay shut with what the society, the professionals or…the community are telling.”* |
| **Caregivers as advocates for service development** | *PCP2, caregiver and NGO representative, PAHO* | *“Things that we do with parents, we also train them to help their children at schools, now we are for example like support, I have developed this idea of parent tutor, or parent mentor, is not only, I mean, when you don’t have a child with a disability, you’re just a parent and parent by law, what does that mean, that you care for eating, dressing, have vaccines, go to school, those are things that parents have to do with any children, yes? But if you have a child with a disability, you take further steps and you’re not only a parent, you’re also a mentor, a tutor, if you’re not a tutor, that will not work and that is linked with your question, like how parents, absolutely, that’s the idea, you don’t have parents that other than parents they are tutors, you won’t have the same results, ever, so you have to give the parents the tools to become good tutors for children with disabilities…many times when they are very young parents, young I mean, recently diagnosed, they generally mothers that generally cry…and you have to be there, holding hands and giving hope and all that only a mother can give to another mother, and there’s no physician that can replace a mother talking to another mother. There’s no way that that can be replaced, no way, no way.* |
|  | *PCP11, researcher, PAHO* | *“From the neighbourhood of the parents we were in touch with, their children go from institution to institution that accept them because the law says so but they don’t have the means how to teach them.”* |
|  | *PCP12, researcher, AFRO* | *“We don’t have very good data on children with disabilities in the provinces, there’s some general population census data but then it’s categorised all people with disabilities and not necessarily reflective of young children with developmental disabilities so we don’t have a database we can work from, we basically go to the community and we ask them to identify families.”* |
| ***The role of advocacy in initiating support*** |  |  |
| ***Actors in advocacy*** |  |  |
| *Personal connections* | *PCP14, caregiver and NGO representative, AMERICAS* | *“The secretary of education in my city is my cousin so…they built a programme called Parents Cope for inclusion parents that have doubts, once a month. So they get each other together and they give a speech and then they work on problems that family has of inclusion at school.”* |
|  | *PCP23, international organisation representative* | *“We pitch it [the intervention] depending on what we think are the opportunities, we are very opportunistic about where we do the work…So we pitch it, we emphasise the elements that could add value to a certain sector…We were visiting a country where one and a half million people are very poor, having problems meeting pay rolls for their civil servant right now, there is a dramatic economic crisis in the country right now. But. But they are interested in autism, right. And the reason is because the…there’s autism in the [family of key members of the government and ruling bodies] and there’s autism in the family of the vice prime minister.”* |
|  | *PCP23, international organisation representative* | *“When I first started doing this work, when I go to a country, I have a routine that I drop in UNICEF offices and try to meet the local country officers, and oftentimes I have to make two separate appointments to see the person responsible for early childhood development and the person responsible for disability.”* |
|  | *PCP10, caregiver and advocate, AMERICAS* | *“On the level where we work, we emphasise to the state what they have to do and we promote that the university does research on the social and moral education for autism, and their rights, and also we are looking for children who are not found, there’s a lot of underdiagnosis. Look, in our country, things work through other people. I talked about CST on a TV channel, in a journal, on Twitter, in a Facebook story, so that other people, stakeholders, like the politicians react to it. We have to be very persistent to get to them.”* |
|  | *PCP129, caregiver and advocate, WESTERN PACIFIC* | *“The government accepted it because they know it is something that World Health Organisation works with, Autism Speaks did it, these are big international organisations and they must know what they are doing exactly so it’s acceptable.”* |
|  | *PCP2, caregiver and advocate, AMERICAS* | *“When public policies have to be created, there are many areas intervening. And other areas don’t understand disability. Disability is like something, you know, it’s not important, but there are labour things and economic things and education and other things. So if you don’t show how this has an impact in education, this has impact in health in your economy, you won’t progress in asking for intervention in disability. You have to show that.”* |
| *Awareness raising* | *PCP4, clinician, AMERICAS* | *“Our mission is improving quality of life of people with autism spectrum conditions and their families and we try to help out and achieve our mission for big strategic lines that we have in the NGO which are on the one side awareness, so we do a lot of awareness.”* |
|  | *PCP4, clinician, AMERICAS* | *“Ideally, like from a research framework, obviously would love to have like evidence-based intervention, but the truth is that life eats you up and you actually end up using first, like what opportunity brings in life.”* |
|  | *PCP11, researcher, AMERICAS* | *“In the neighbourhoods we work in, the trainings for parents are done by civil society, so these trainings make access a bit more free for parents and they are better developed specifically for this population.”* |
|  | *PCP14, caregiver and NGO representative, AMERICAS* | *“So when I go to the city mayor, he looks at me [and says] WHO? And when I go to the families, they look like, she is helping us, so let’s help her. So there is this, they need to see the face. Not that I’m strong, I’m just going there... But I think in our country it’s very important to have the face. Even though it’s not from personality but I felt that it’s important to have a face. So that’s why I think it’s working here because there is a face to the programme.”* |
|  | *PCP123, clinician, SOUTH-EAST ASIA* | *“So for us it was the decision was to go for a programme that is not bound for copyright. That would allow more accessibility to the people of a country.”* |
|  | *PCP12, researcher, AFRO* | *“[We were looking for] a training that we don’t have to pay for training, that it is open access materials so we don’t have to pay for every new booklet or every information and another thing is reaching fidelity, with many other interventions it takes a long time and it’s a very difficult process to reach fidelity as a facilitator or trainer.”* |
|  | *PCP23, international organisation representative* | *“The UN’s main interest in this opportunity was actually related to sexual violence. In this country, children with development disability, almost every week, sexual violence, you know, rape, in many cases because the way the legal system is set up there, is that there’s no witness, often the children are killed after cases recorded. So UN was interested in looking at this issue from a sexual violence, with disability perspective. Because this is very the government’s interests are, even though the UN agencies are saying that we should look at more from a gender violence perspective.”* |
| *Caregiver experiences* | *PCP2, caregiver and advocate, AMERICAS* | *“This is a book we have done with people with autism and it is remunerated work with people with a reading disability, so I generally hire and pay youth with autism and have a great time. And they love it.”* |
|  | *PCP23, International organisation representative* | *“The major problem in [a country in AFRO region] is that… almost every week, here’s this thing about a child with a developmental disability or disability…sexual violence, you know, rape, so on and so forth, in many cases because the way the legal system is set up there, is that there’s no witness, there’s no case, often the children are killed after cases recorded.”* |
|  | *PCP14, caregiver and NGO representative, AMERICAS* | *“We decided we had to make a foundation and my husband, he is very you know strategic and financial man, so he told me don’t build something that every time have to put money on it. So I start my research to find something that I can afford the introduction, afford to maintain, but don’t need to put money every single month.”* |

| ***Using evidence to drive advocacy*** |  |  |
| --- | --- | --- |
|  | *PCP19, trainer of master trainers, EASTERN MEDITERRANEAN REGION* | *We need to be equipped with the data also to prove to the parents and the facilitators and everyone that look, the data says that it worked.* |
|  | *PCP202, clinician and researcher, AMERICAS* | *“If you decide to go through a whole cross-cultural adaptation, you still don’t know what is effective in the intervention, to know it, you will have to do a randomised control trial, again.”* |
|  | *PCP5, clinician, SOUTH-EAST ASIA* | *“People are not used to doing a lot of documentation and filling in forms about what you think this is and that is, and in our country that’s not our culture at all, so people go to the doctor, you get your diagnosis and you are out…so for this pilot phase, people felt very obliged because they are part of this programme but still…they couldn’t know what to say…we are quite worried what will happen with more kind of lower socio-economic status families...”* |
|  | *PCP202, clinician and researcher, AMERICAS* | *“The problem is, you cannot do randomised control trial forever, right? Is very expensive…”* |
|  | *PCP14, caregiver and NGO representative, AMERICAS* | *“I follow the order of rules. And the rule is that I have to have data collection. But for me personally is more important to get this families together then to have data, but, but since I’m a follower of rules, I know that I have all the steps to that I can prove. So for me it’s okay.”* |
|  | *PCP22, international organisation representative* | *I think that’s just the world we live in, when you show that this is effective, we may be willing to put money towards this but we can’t afford to put money towards something that is not yet evidence-based.* |
|  | *PCP121, caregiver and advocate, AMERICAS* | *“There is a point that we need local statistics, we need the local, because we live there.”* |
|  | *PCP12, researcher, AFRO* | *“In low-and middle income countries people definitely do ask the question why do you need to do research, why can’t you just implement the programme and scale it up quicker, because there’s such a great need, what will research tell us anything more than you know it’s okay, it’s benefitting parents and families.”* |
|  | *PCP128, researcher, AFRO* | *“I always feel like there is an expectation of evidence base but when you go and look at your own context and see there’s no data and interventions then…it’s very difficult to actually transfer and say that it is evidence-based. Yes, it might be in another country in another setting.”* |
|  | *PCP2, caregiver and advocate, AMERICAS* | *“And then we had this meeting, lot of people…and when a researcher started to explain the statistics, people were almost asleep…and then it was my turn. And everybody woke up. Because numbers and I don’t get along…so I started to explain statistics on an everyday basis. And I said look at this pie. Don’t look at the numbers. Look at the pie. Look at the slice, it’s very big. So this means that children are at home. This means that, so if children are at home, what do we have to do.”* |
|  | *PCP12, researcher, AFRO* | *“We don’t have very good data on children with disabilities in the provinces, there’s some general population census data but then it’s categorised all people with disabilities and not necessarily reflective of young children with developmental disabilities so we don’t have a database we can work from, we basically go to the community and we ask them to identify families.”* |

# Using evidence to drive advocacy

| Evidence is associated with research |  |
| --- | --- |
| PCP101 | That someone has done RCT? |
| PCP122 | That it has been proved before…to be…effective. |
| PCP129 | You have the data. Backed by data. Survey data. |
| PCP127 | Clinical data. Empirical data. |
| PCP205 | And had proof it works. |
| PCP19 | We are in a time when we have statistical capabilities, it’s still tricky when it comes to mental health, but I mean we can measure the behaviour, we can measure communication, that is measurable, so I think measuring those aspects after the whole programme was taken and of course during, over a longer period, that is how evidence based is. |
| Evidence is deducted from a well-known platform or brand |  |
| PCP123 | Or that we heard like four presentations at INSAR about? |
| PCP9 | If you say something is evidence-based, I would expect that there is a research paper talking about that and whether something works is totally based on empirical evidence. So you must have a published paper saying, or giving the details of what was being done, how it was being tested, and we have to look at papers ourselves and see if it’s valid research. |
| PCP6 | It is [an intervention] based on JASPER and other kind of programmes, I think it is evidence-based of course. It is coming from you know programmes that have a lot of peer reviewed journals, showing that the programme can bring about a significant change in the outcomes. |
| PCP20 | The trainers told us that it’s mostly based on ABA as well as JASPER, and so because we have read a lot of papers and some articles about these two therapies so I think it’s quite evidence-based. |
| Evidence is something others have experienced |  |
| PCP121 | Lots of families went through that before me, that’s what I would think. |
| Evidence is a guarantee and enables one starting the programme |  |
| PCP3 | It might give the guarantee that it will work and you can get benefit out of it and good results and outcomes, so it might give you more confidence to start running it. |
| Evidence is when a child’s symptoms improve |  |
| PCP10 | Evidence is that the child can get better, how the child can get better. So if I do an intervention many times and have no idea and then finally I understand what is happening, and then when the child shows, then it’s evidence that he learnt the intervention. |
| PCP6 | Effective in a sense that I can see a significant change or observable change in parents’ attitudes and skills and mindset in supporting their children. |
| Evidence can be deducted from psychological concepts accepted as valid |  |
| PCP123 | I think we also look at the underlying principles and if they are evidence-based, there could be many programmes that focus on imitation and joint attention and we know that those work. |
| PCP6 | It is [an intervention] based on JASPER and other kind of programmes, I think it is evidence-based of course. It is coming from you know programmes that have a lot of peer reviewed journals, showing that the programme can bring about a significant change in the outcomes. |
| Levels of evidence |  |
| PCP101 | I think it’s like a hierarchy…if you have one [intervention] with a big RCT with a lot of people which is local that’s…but then if there’s someone somewhere that showed that it works, it’s better than nothing…so of course the more evidence, the better. |
| Evidence with different meanings for stakeholders |  |
| PCP22 | The master trainers that I’ve trained, the feedback that I’ve heard from them is that they are very excited by the changes that they see in these kids, from home visit to home visit, for many of them, that’s all the data they need, cause they’re seeing changes on a child, on a child basis. But in the research community, it’s not gonna work for an academic, no one is gonna fund it because 5 kids made changes, but it’s enough to keep those MTs passionate about the programme and pushing for it to happen. |
| PCP23 | I don’t get that sense that there is a reciprocation at that policy maker level, in terms of whether they can differentiate what is evidence base, what is evidence informed, Or even different levels of rigorousness. |
